# Stimulation of Osteogenic Differentiation of Induced Pluripotent Stem Cells (iPSCs) Using Bioactive Glasses: An *in vitro* Study

**DOI:** 10.3389/fbioe.2019.00355

**Published:** 2019-11-22

**Authors:** Saeid Kargozar, Nasrin Lotfibakhshaeish, Somayeh Ebrahimi-Barough, Bahareh Nazari, Robert G. Hill

**Affiliations:** ^1^Tissue Engineering Research Group, Department of Anatomy and Cell Biology, School of Medicine, Mashhad University of Medical Sciences, Mashhad, Iran; ^2^Department of Tissue Engineering and Applied Cell Sciences, School of Advanced Technologies in Medicine, Tehran University of Medical Sciences, Tehran, Iran; ^3^Department of Medical Biotechnology, School of Advanced Technologies in Medicine, Tehran University of Medical Sciences, Tehran, Iran; ^4^Unit of Dental Physical Sciences, Barts and the London School of Medicine and Dentistry, Queen Mary University of London, London, United Kingdom

**Keywords:** bone tissue engineering, osteogenesis, induced pluripotent stem cells, bioactive glass, *in vitro*

## Abstract

Selection and use of an optimal cell source for bone tissue engineering (BTE) remain a challenging issue; the invention of induced pluripotent stem cells (iPSCs) have created new hopes on this regard. At the present study, we attempted to show the usability of iPSCs in combination with bioactive glasses (BGs) for bone regeneration applications. For this aim, iPSCs were cultured and incubated with the strontium and cobalt-containing BGs for different intervals (1, 5, and 7 days). The cell cytotoxicity and attachment were assessed using MTT assay and scanning electron microscopy (SEM), respectively. Moreover, the osteogenic differentiation of iPSCs seeded onto the glasses was evaluated using alkaline phosphatase (ALP) activity assay and real-time PCR. The obtained results clarified that although the cell viability is decreased during a 7 day period, the iPSCs could adhere and expand onto the BGs particles and over-express the osteogenic markers, including osteocalcin, osteonectin, and Runx2. Based on the data, we conclude that iPSCs in a combination of BGs can be considered as a potential candidate for BTE strategies.

## Introduction

Considering the high prevalence of bone injuries across the globe, the need to develop and optimize novel therapies are of great importance among researchers of different fields like materials scientists, biologists, and clinicians (Amini et al., 2012). On this matter, a broad range of biomaterials, cells, and growth factors, i.e., building blocks of the tissue engineering field, have been developed and used (Winkler et al., 2018; Miola et al., 2019). It is worth mentioning that although the results of conventional grafts (auto, allo, and xeno) have been beneficial, there are a couple of limitations in front of their extensive use for bone replacement, for example, shortage of donors, immunological rejection, and disease transmission (Oryan et al., 2014).

Up to now, a large number of materials, including ceramics, have been applied for treating bone-related damages and injuries (Denry et al., 2016; Fernandes et al., 2017; Atkinson et al., 2019). Among them, bioactive glasses (BGs) are considered as promising substitutes in bone tissue engineering (BTE) with a long history in clinical setting because of their excellent properties as follow; (1) the ability to attach to the bone tissue; (2) improving osteoblasts proliferation; (3) rendering antibacterial activities; and (4) promoting angiogenesis (Johari et al., 2016; Kargozar et al., 2017a, 2018a,b,c; Baino et al., 2018; Kargozar et al., 2019a). Apart from these features, BGs could be combined with other materials like bio-polymers to increase their regenerative potential as well as overcome some of their inherent limitations like brittleness. Most of the biological activity of BGs is related to releasing various therapeutic ions form their structure to the adjacent environment (Kargozar et al., 2018c). Nowadays, new formulations of this synthetic material developed to improve its biological activities, which is in favor of regenerative medicine strategies. As an illustration, Kargozar et al. in 2017 succeeded to improve the osteogenesis and angiogenesis activities of BGs using the incorporation of strontium and cobalt into the glasses structure (Kargozar et al., 2017b).

Although the results of previously performed studies are absolutely in agreement with the efficacy of BGs in bone regeneration *in vivo*, the healing process is accelerated when stem cell-seeded glasses implant (Jing et al., 2018; Kargozar et al., 2018d).

As well-documented, cells with different origins have a critical role regarding the acceleration of bone healing process (Kargozar et al., 2019b). Cells (somatic and stem cells) imply their restorative roles through differentiation and replacement of injured tissue as well as secreting various therapeutic bioactive molecules (da Silva Meirelles et al., 2009). Up to now, varieties of stem cells have been used for bone regeneration applications including adult, fetal, and embryonic stem cells. However, there are critical limitations in the case of their use for regenerative medicine, including source constraint, immunorejection, the possibility of transmitting disease, and risk of carcinogenicity (Choumerianou et al., 2008; Poulos, 2018).

After being discovered by Yamanaka's group in Kyoto University (Okita et al., 2007), much interest has been paid on the use of induced pluripotent stem cells (iPSCs) for the repair and regeneration of damaged tissues like the bone. These cells show high potential regarding proliferation and differentiation without eliciting immunological responses, which are counted as their merits in comparison to other type cell sources (Lou, 2015). Moreover, iPSCs could differentiate into all specialized cell types residing in the bone tissue under a proper condition (Jeon et al., 2016). Thus, iPSCs offer a proper and rich source of bone-forming osteoblasts, which could ultimately generate osteogenic cells (mesenchymal stem cells (MSCs), osteoblasts, or osteocyte-like cells) (Zhu et al., 2019).

In the present study, we showed the osteogenic potential of iPSCs for bone regeneration applications through their culturing onto strontium (Sr)- and cobalt (Co)-substituted BGs that previously synthesized and characterized (Kargozar et al., 2016). Although osteogenic differentiation of iPSCs were evaluated in contact with some types of ceramics (e.g., akermanite and bredigite) (Dong et al., 2016; Chen et al., 2017), to the best knowledge of the authors, it is the first report on the applicability of simultaneous use of iPSCs and BGs regarding BTE applications, which can be considered a pioneer work in this important field. With respect to the promising features of both BGs and iPSCs, it could be interesting to evaluate their combination for bone reconstruction.

## Materials and Methods

### Synthesis and Characterization of BGs

As well-described in previous work (Kargozar et al., 2016), the melt-derived BGs were synthesized based on a multicomponent system (see Table 1). Briefly, the components were placed in an electric furnace (Lenton, Hope Valley, UK) to melt at 1,400°C for 90 min. After finishing the melting process, the glasses were quickly quenched by water, and then the obtained frits were ground and sieved to make glass particles with a size of <38 μm. The results of X-ray diffraction (XRD) and Fourier-transform infrared spectroscopy (FTIR) confirmed the glassy state of the synthesized samples before immersion in simulated body fluid (SBF) and proved their bioactivity through the formation of hydroxyapatite -like layer on their surface after immersion in SBF.

**Table 1:** The composition of the BGs (mol %) used for stimulating iPSCs osteogenesis (Kargozar et al., 2016).

**Sample**	**SiO_**2**_**	**P_**2**_O_**5**_**	**CaO**	**SrO**	**Na_**2**_O**	**MgO**	**K_**2**_O**	**CoO**
Sr	41.2	5.06	30.14	6	7.17	3.26	7.17	0
Ca-Co	41.2	5.06	35.64	0	7.17	3.26	7.17	0.5
Sr-Co	41.2	5.06	29.64	6	7.17	3.26	7.17	0.5

### iPSCs Culture

**Figure 1:**
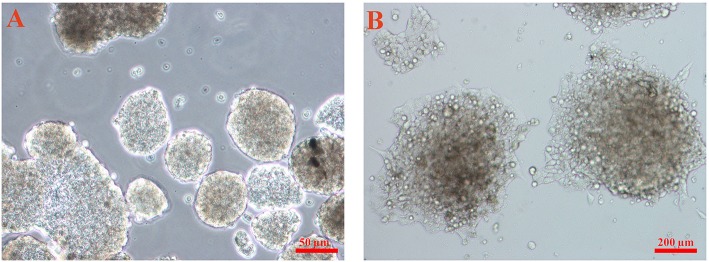
The inverted microscopy image of the iPSCs used for the experiments. **(A)** The cells cultured in tissue culture plates (TCPs) and **(B)** cultured on the feeder layer.

The iPSCs derived from human fibroblasts (hiPSCs) purchased from the cell bank of Stem Cells Technology Research Center (Tehran, Iran) were previously used in another study of the authors (Hoveizi et al., 2017) (Figure 1). After transferring to the laboratory, the cells were placed in the culture Petri dishes coated with mouse embryonic fibroblast (MEF) cells were inactivated by mitomycin C and incubated in at 37°C in a humidified atmosphere of 5% CO_2_. Until the day of the test, the cell culture medium, DMEM/F12 containing 10 ng/ml bFGF supplemented with fetal bovine serum (FBS), penicillin/streptomycin, and non-essential amino acids (all reagents from Gibco, USA), was replaced every 2 days.

### Cell Viability Assessment

The effect of the glasses on the growth and proliferation of hiPSCs was evaluated using the standard colorimetric 3-(4, 5-dimethyl-2 thiazolyl) - 2,5-diphenyl- 2H-tetrazolium bromide (MTT) assay. For this purpose, the cells were seeded into 96-well cell culture plates (SPL Life sciences, Korea) at a concentration of 5 × 10^3^ cells/ well. After the incubation of 24 h, the culture medium of iPSCs was changed with the conditioned media (containing 4 mg/ ml of each glass), and cells were cultured in this condition for 1, 5, and 7 days. At the end of each time point, the MTT solution (5 mg/ml) (Sigma-Aldrich, USA) was added to the plates and incubated for another 4 h. Then all the cell culture medium was pulled out, and dimethyl sulfoxide solution (DMSO) (Sigma-Aldrich, USA) added to the plates. As a final point, optical density (OD) of each plate was measured by a spectrophotometer (Synergy HT, BioTek, USA) at 570 nm wavelength. The number of living cells was calculated using the following formula:

$$\begin{array}{l} Viability=\frac{\text{the mean absorbance of the sample}}{\text{the mean absorbance of the control}}\;\times 100 \end{array}$$

### Adhesion Evaluation of the iPSC on the BGs

In order to observe the morphology of the grown iPSCs onto the glasses surface, scanning electron microscopy (SEM) was performed. For this aim, the cells-seeded BGs were firstly washed with phosphate-buffered saline (PBS) and then fixed in a series of solutions including 2.5% (v/v) glutaraldehyde (Merck, Germany) in 0.1 M PBS (2 h) and 0.1% (v/v) osmium tetroxide (OsO4) (Sigma-Aldrich, UK) in 0.1 M PBS (30 min). In the next step, the fixed samples were dehydrated using graded acetone series (30, 50, 75, and 100%) and maintained in 100% acetone before freeze-drying (BOC Edwards, Crawley, UK). At the final step, the cell-glass samples were sputter coated with gold and viewed SEM (Tescan, Vega ts5136MM, CZ) at accelerating voltage of 15 keV.

### Alkaline Phosphatase (ALP) Activity

The osteogenic potential of iPSCs cultured onto the BGs was evaluated using the measurement of ALP levels secreted by the cells. To do this assay, about 1 × 10^5^ cells were seeded onto the glasses and incubated up to 21 days. The same cells were cultured in 24-well plates as a control group (2 D culture). The supernatant of the cultures was collected at days 1, 7, 14, and 21 and tested for the ALP activity. According to the manufacture protocol (Pars Azmoon, Iran), 800 μl of diethanolamine mixed to 200 μl of para-nitrophenyl phosphate, and then the obtained solution was added to 20 μl of the supernatants. Finally, the mixtures were introduced to the spectrophotometer (Synergy HT, BioTek, USA), and OD of samples was recorded at 570 nm wavelength.

### RNA Extraction and Gene Expression Evaluation

To survey the osteogenic differentiation of iPSCs in contact with the BGs, the expression level of specific genes involved in osteogenesis was measured. On this object, the iPSCs were cultured in the conditioned media (containing 4 mg/ml of the glasses) for 21 days. After completing the period, total RNA of the iPSCs treated with the conditioned media was isolated by the RNAeasy Mini Kit (Qiagen, USA). To ensure a lack of genomic DNA contaminations of RNA preparations, all the samples were treated by DNAse I amplification grade (Invitrogen). Next, single strand cDNA, needed for performing quantitative PCR (qPCR), was synthesized from the isolated RNA by a PrimeScript 1st strand cDNA synthesis kit (Takara, Japan). Then the assay was carried out using the specific primers for genes of GAPDH (homeobox gene MSX2), BGLAP (osteocalcin), ISBP (bone sialoprotein 2), and Runx 2 (runt-related transcription factor 2) (Table 2). Rotor-Gene™SYBER®Green PCR Kit (Qiagen, USA) was used to conduct qPCR assay using the Rotor-Gene 6000 Real-Time PCR Machine (Qiagen, USA). The thermal cycling conditions comprised an initial denaturation step at 90°C for 10 min, followed by 40 cycles at 90°C for 30 s, 60°C for 30 s and 72°C for the 30s. All the experiments were performed in duplicate for each sample and time point. The 2^−ΔΔCt^ method was applied to calculate relative quantification in the expression of genes. All the fold changes in gene expression were normalized to GAPDH.

**Table 2:** The characteristic of primers used for real-time PCR assay.

**Primer name**	**Forward primer sequences**	**Length (bP)**	**Reverse primer sequences**	**Length (bP)**
BGLAP	GGTGCAGCCTTTGTGTCCAAG	21	AACTCGTCACAGTCCGGATTGAG	23
IBSP	GATTTCCAGTTCAGGGCAGTAGTG	24	GTTTTCTCCTTCATTTGAAGTCTCCTC	27
Runx2	ACTCTACCACCCCGCTGTCTTC	22	AGTTCTGAAGCACCTGCCTGG	21
GAPDH	TCGCCAGCCGAGCCA	15	CCTTGACGGTGCCATGGAAT	20

### Statistics

All the quantitative data obtained from the MTT test and ALP activity assay were statistically analyzed using one-way ANOVA test followed by Tukey's *post hoc* test to determine whether there are any significant differences. The *p*-value of 0.05 (*P* ≤ 0.05) was considered to be statistically significant.

## Results

### Physicochemical Characteristics of the Glasses

**Figure 2:**
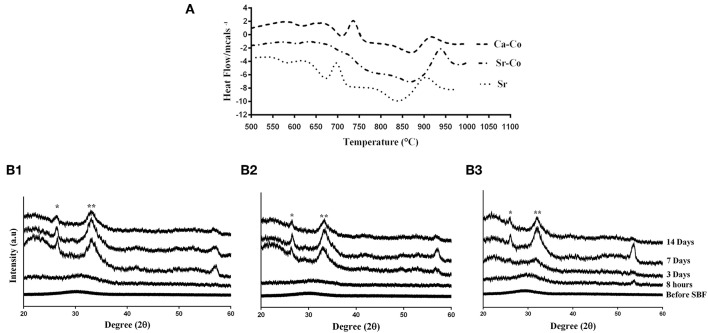
Data related to physico-characterization of the as-prepared BGs. **(A)** Glass transition temperature (Tg) of the glass samples determined by differential scanning calorimetry (DSC) analysis. **(B1–B3)** XRD spectra of the samples (Sr-, Ca-Co, and Sr-Co glasses, respectively) before and after incubation in SBF. With some modifications from Reference (Kargozar et al., 2016). ^*^ and ^**^ are related to the peaks of the formation of silica gel and hydroxyapatite like layer at 2θ = 26° and 2θ = 32°, respectively.

As well-reported in our previously published paper (Kargozar et al., 2016), all the BG samples are in an amorphous state before immersion into SBF, while the formation of a hydroxyl apatite-like layer observes after incubation in SBF. Although the incorporation strontium and cobalt into the glass structure were successfully performed, any significant adverse effect on the bioactivity of the glasses did not observe, confirmed by XRD, FTIR, and SEM images (see Figure 2).

### Cell Viability Assay

**Figure 3:**
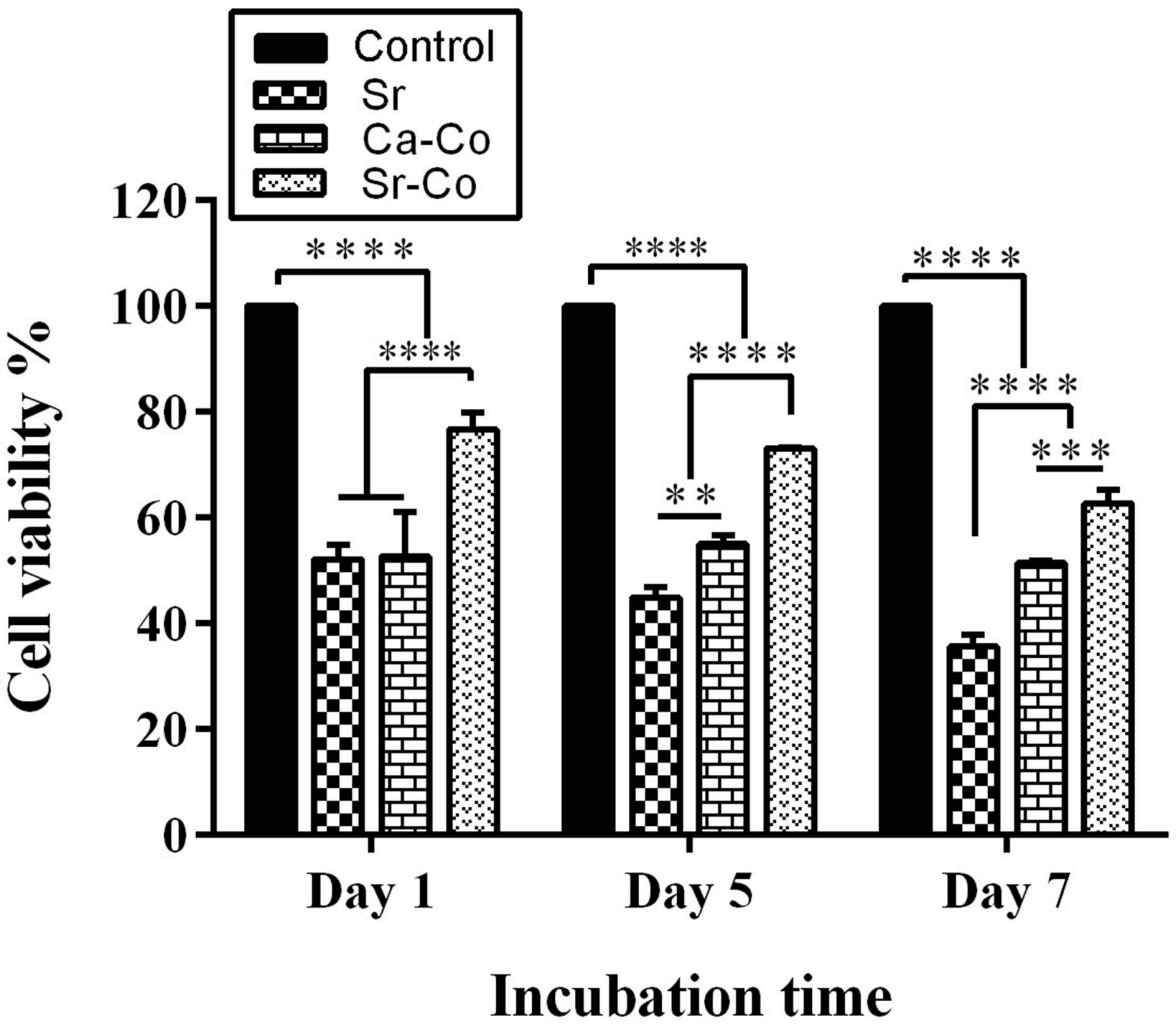
The graph representing the viability percentage of the iPSCs cultured in the 2D system (control group) and onto different glass samples after at days 1, 5, and 7. ^*^p < 0.05, ^**^p < 0.01, ^***^p < 0.001, and ^****^p < 0.0001.

The results obtained from the viability assay showed that the glass samples cause a significant decrease in cell growth and proliferation of the iPSCs. As shown in Figure 3, the samples had an inhibitory effect on cell viability within the incubation times. This inhibition is more obvious over time; the percentage of cell viability was 55, 57, and 73 % for the Sr, Ca-Co, and Sr-Co in day 1 while these amounts were 37, 52, and 65% for their counterparts in day 7.

### Cell Attachment Study

**Figure 4:**
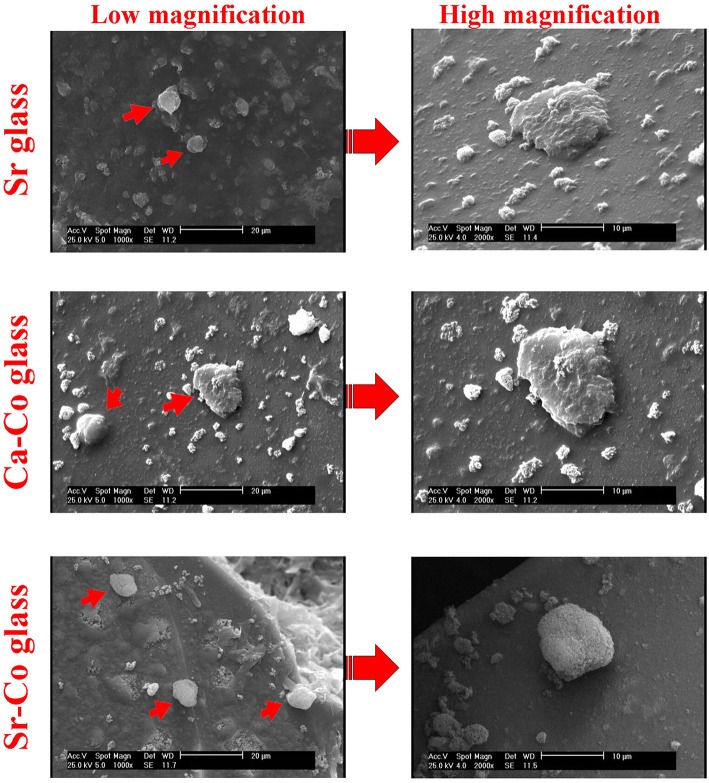
The SEM images captured from the iPSCs onto the glass samples after 5 days post-seeding. As can be seen, the cells maintain their round morphology on the surface of the glass samples.

The SEM micrographs revealed the iPSCs could easily attach to the surface of the glass samples after the cell seeding process. As shown in Figure 4, the cells expand and keep their native morphology on the glasses' surface after 5 days of incubation.

### ALP Activity Assessment

**Figure 5:**
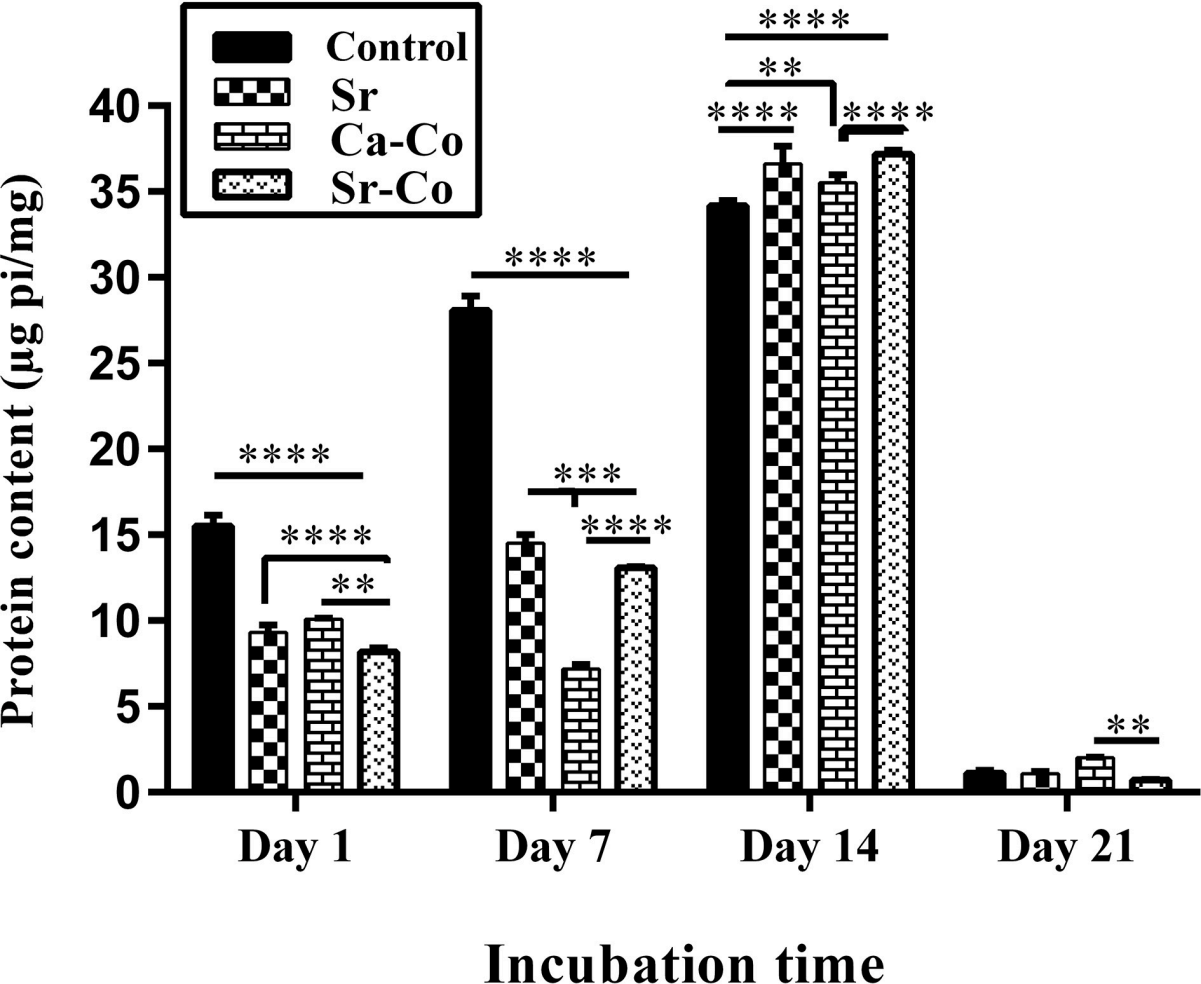
The graph showing the amounts of ALP produced by iPSCs after culturing onto the glass samples. ^*^p < 0.05, ^**^p < 0.01, ^***^p < 0.001, and ^****^p < 0.0001.

The results of ALP activity assay is shown in Figure 5. ALP is a well-defined marker of differentiation and mineralization of osteoblast cells, which increased by enhancing osteoblast activity. The recorded values of ALP in the groups CNT, Sr, Ca-Co, and Sr-Co are 15.53, 9.45, 10.1, and 8.25 μg Pi/mg, respectively in day 1. All the amounts show a significant increase in day 14 as follows 34.35, 37.05, 35.61, and 37.37 μg Pi/mg in the case of CNT, Sr, Ca-Co, and Sr-Co groups, respectively. However, a significant decrease in the ALP production was observed at day 21 post-incubation so that all the records related to the production of ALP were below 2 μg Pi/mg.

### qRT-PCR

**Figure 6:**
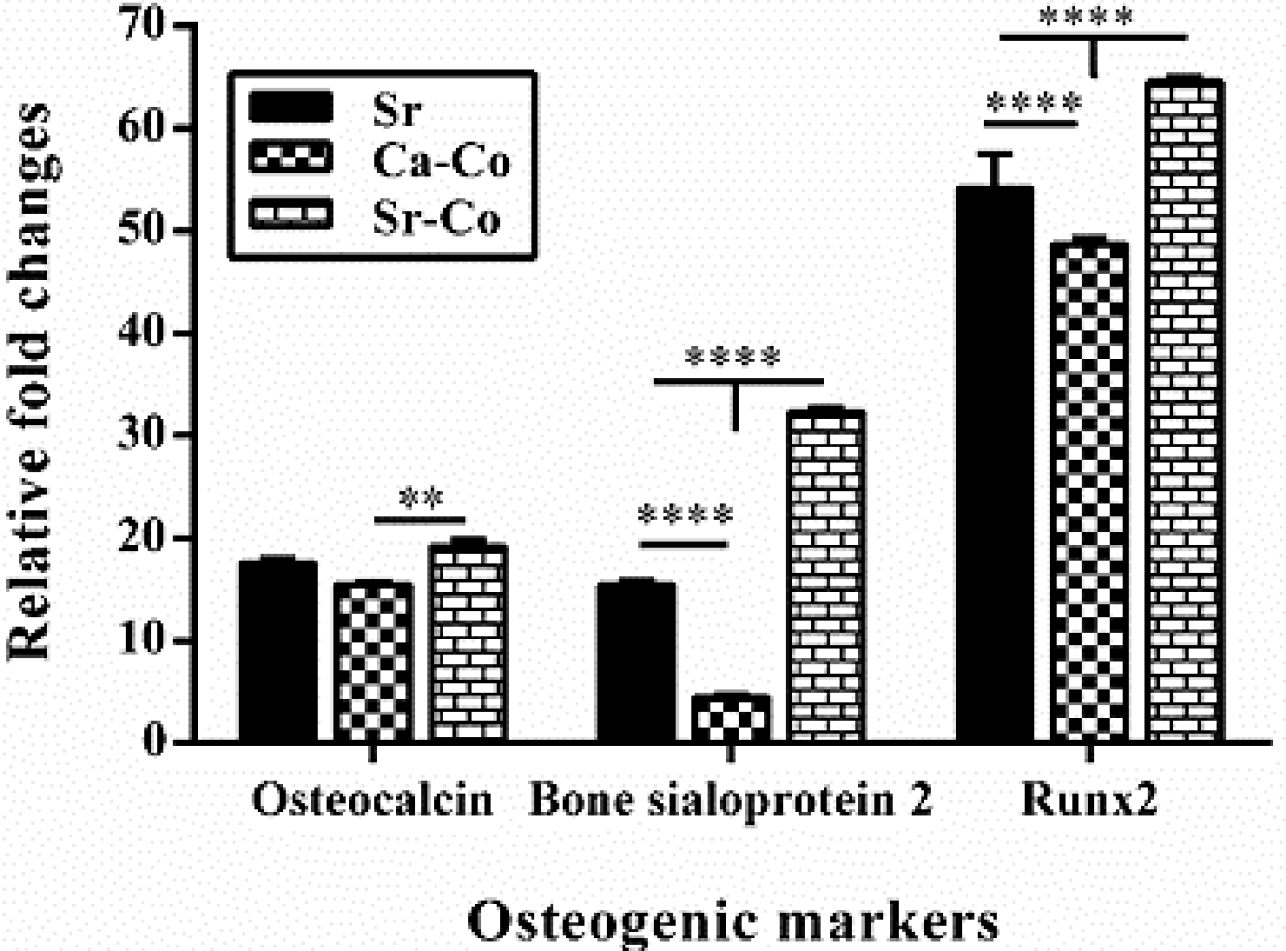
The results obtained from qRT-PCR showing the effect of the BGs on the expression of osteogenesis markers after 21 days' post-incubation. The data were evaluated in triplicate and collected from three independent experiments. The mRNA expression levels were normalized to GAPDH in each cell line. ^*^p < 0.05, ^**^p < 0.01, ^***^p < 0.001, and ^****^p < 0.0001.

The osteogenic potential of the iPSCs using culturing onto the glasses at molecular levels was determined by qRT-PCR using ΔΔCt method. As shown in Figure 6, the expression of osteogenesis-related genes (osteocalcin, bone sialoprotein 2, and Runx 2) were up-regulated in the cells cultured onto the glass samples as compared to the conventional condition (cell culture plates) after 21 days. The amounts of up-regulation of osteocalcin was recorded as 17.43, 15.56, and 19.16 relative fold changes for the Sr, Ca-Co, and Sr-Co groups, respectively. In the case of bone sialoprotein 2, the records were 15.4, 4.55, and 32.66 for the Sr, Ca-Co, and Sr-Co groups, respectively. The highest expression was related to Runx2 in the groups; 54.7, 48.8, and 64.53 in the case of the Sr, Ca-Co, and Sr-Co groups, respectively. Therefore, the highest expression of the osteogenic genes is related to the group glass Sr-Co.

## Discussion

The use of iPSCs for bone healing applications is one of the most interesting research topics in the field of tissue engineering and regenerative medicine. These cells offer new opportunities for repair and regeneration of the bone, which is not achievable in the case of other cell sources (e.g., embryonic stem cells) including the lack of immune rejection along with the ability to differentiate into osteogenic cells (Lou, 2015). As an illustration, Zhu et al. have recently demonstrated the moue derived iPSCs could successfully differentiate to osteoblast lineage cells (Zhu et al., 2019). The combination of these cells with various natural and synthetic materials have been evaluated in terms of BTE (Ardeshirylajimi, 2017). However, there is a gap in the literature regarding their usability when applied with BGs. In this study, we evaluated the osteogenic potential of iPSCs in contact with Sr- and Co-substituted BGs, which were well-characterized in our previously published work (Kargozar et al., 2016). BGs are identified as potent substances in tissue engineering strategies with a long history in bone reconstruction applications (Baino et al., 2018). Releasing therapeutic ions form their structure into the biological environment results in stimulation of cells to osteogenic differentiation both *in vitro* and *in vivo*. It has been well-clarified that Sr^2+^ ions could improve osteogenesis via two distinct routes, i.e., the induction of osteoblastic activity and reduction of osteoclastic activity. On the other hand, Co^2+^ has an important effect on improving angiogenesis, which could be useful for accelerating the new bone formation and thereby bone healing process (Wu et al., 2012).

As the toxicity of any biomaterials should be considered before further *in vivo* evaluations, the iPSCs were cultured onto the glasses, and the results were recorded during 1, 3, and 7 days post-cell seeding. As shown in Figure 3, the glasses had a significant inhibitory effects on the cell viability in different incubation time. The percentage of the cell viability is decreased over the time; from 55, 57, and 73 % in the case of the Sr, Ca-Co, and Sr-Co groups in day 1 to 37, 52, and 65 % for the same groups in day 7. The reason for this reduction might be related to cell apoptosis during the incubation of the iPSCs with glass samples; however, there need more specific evaluations to determine the molecular mechanisms involved in the reduced cell viability. It is worth mentioning that most of the previously performed studies report cyto- and bio-compatibility of different formulations of BGs, making them excellent candidates for bone regeneration strategies (Wilson et al., 1981; Kargozar et al., 2019c). The images obtained from SEM (see Figure 4) showed the good attachment iPSCs onto the BGs, confirming the cytocompatibility of the samples. It has been previously reported that various formulations of BGs are a suitable substrate for the adhesion and expansion of mammalian cell lines (Ojansivu et al., 2018).

The obtained results of ALP activity assay showed the iPSCs could produce ALP when incubated with the glass samples. The reason of this improvement is related to this fact that the ions released from BGs (e.g., Si^4+^, Ca^2+^, Sr^2+^) could up-regulate the genes related to the bone formation such as osteocalcin (Jell et al., 2006). As shown in Figure 4, the best results belong to the control group (2D culture system) up to day 7 of culturing (28 μg Pi/mg); however, the production of ALP is higher in the cells cultured onto the glass samples at day 14 (the Sr-Co group: 37.5 μg Pi/mg). The results are consistent with the previous findings, i.e., the positive effect of ions released (e.g., Sr^2+^) from the BGs on osteogenesis via the induction of osteoblast markers like ALP (Oh et al., 2010). However, the amounts of ALP production was meaningfully decreased in day 21, which are controversially to interpret as the decrease of ALP production is observed when osteoblasts differentiate to osteocytes. The data obtained from q-PCR also confirms the positive effects of the BGs on osteoblastic differentiation of iPSCs at the molecular level. As shown in Figure 6, the expression of all the genes involved in osteogenesis (osteocalcin, bone sialoprotein, and Runx2) was up-regulated at 21 days' post incubation. However, the best results belong to the group treated with Sr-Co contacting glasses, representing the simultaneous effect of Sr and Co ions regarding osteogenesis process. The results are consistent with our previous work in which genes involved in osteogenesis was up-regulated in SaOS-2 cells cultured in a conditioned media (RPMI-1640 containing 4 mg/ml of the same glasses) (Kargozar et al., 2017b).

## Conclusion

The use of iPSCs for repair and regeneration of bone injuries is at the beginning steps and need more research to understand their potential in this regard. In this study, we evaluated the osteogenic potential of iPSCs in the combination of Sr and Co-substituted glasses for the first time. The results of the cell viability showed that the incubation of iPSCs with BGs could result in a significant reduction in the cell viability percentage; might be related to the cell apoptosis. The data obtained from ALP activity assay and real-time PCR clarified that the BGs could stimulate the osteogenic differentiation of iPSCs. Therefore, this combination is considered an appropriate approach to the treatment of bone damages. However, the evaluation of this strategy should be performed through other well-defined *in vitro* and *in vivo* experiments to identify specific molecular mechanisms involved in the interaction of iPSCs with BGs.

## Data Availability Statement

The raw data supporting the conclusions of this article will be made available by the authors, without undue reservation, to any qualified researcher.

## Author Contributions

SK: writing the first draft as well as funding. NL: revising the manuscript as well as funding. SE-B: figures and tables. BN: performing the molecular assays. RH: revising the manuscript.

### Conflict of Interest

The authors declare that the research was conducted in the absence of any commercial or financial relationships that could be construed as a potential conflict of interest.
